# Supplementary material for: Resolving the full spectrum of human genome variation using Linked-Reads
Source: Genome Res. 2019 Apr;29(4):635–45. doi: 10.1101/gr.234443.118 (PMC6442396; doi:10.1101/gr.234443.118)
Supplement: Supplemental Material [file supp_gr.234443.118_Supplemental_longranger-2.2.zip › longranger-2.2.tar.bz2!/lr2.2-public/lib/stan/cmdstan/stan/doc/api/html/operator__unary__increment_8hpp_source.html]

Stan: src/stan/agrad/rev/operators/operator\_unary\_increment.hpp Source File


|  |  |
| --- | --- |
|  | Stan  2.5.0  probability, sampling & optimization |


- Main Page
- Related Pages
- Namespaces
- Classes
- Files

- File List
- File Members

All Classes Namespaces Files Functions Variables Typedefs Enumerations Enumerator Friends Macros Pages

- src
- stan
- agrad
- rev
- operators

operator\_unary\_increment.hpp

Go to the documentation of this file.

1 #ifndef STAN\_\_AGRAD\_\_REV\_\_OPERATORS\_\_OPERATOR\_UNARY\_INCREMENT\_HPP

2 #define STAN\_\_AGRAD\_\_REV\_\_OPERATORS\_\_OPERATOR\_UNARY\_INCREMENT\_HPP

3

4 #include <stan/agrad/rev/var.hpp>

5 #include <stan/agrad/rev/internal/v\_vari.hpp>

6 #include <boost/math/special\_functions/fpclassify.hpp>

7

8 namespace stan {

9  namespace agrad {

10

11  namespace {

12  class increment\_vari : public op\_v\_vari {

13  public:

14  increment\_vari(vari\* avi) :

15  op\_v\_vari(avi->val\_ + 1.0, avi) {

16  }

17  void chain() {

18  if (unlikely(boost::math::isnan(avi\_->val\_)))

19  avi\_->adj\_ = std::numeric\_limits<double>::quiet\_NaN();

20  else

21  avi\_->adj\_ += adj\_;

22  }

23  };

24  }

25

35  inline var& operator++(var& a) {

36  a.vi\_ = new increment\_vari(a.vi\_);

37  return a;

38  }

39

51  inline var operator++(var& a, int /\*dummy\*/) {

52  var temp(a);

53  a.vi\_ = new increment\_vari(a.vi\_);

54  return temp;

55  }

56

57  }

58 }

59 #endif

boost::math::isnan

bool isnan(const stan::agrad::var &v)

Checks if the given number is NaN.

**Definition:** boost\_fpclassify.hpp:69

stan::agrad::operator++

var & operator++(var &a)

Prefix increment operator for variables (C++).

**Definition:** operator\_unary\_increment.hpp:35

stan::agrad::var::vi\_

vari \* vi\_

Pointer to the implementation of this variable.

**Definition:** var.hpp:40

unlikely

#define unlikely(x)

**Definition:** likely.hpp:9

v\_vari.hpp

var.hpp

stan::agrad::var

Independent (input) and dependent (output) variables for gradients.

**Definition:** var.hpp:27


---

[ Stan Home Page ]

*© 2011–2014,
Stan Development Team.*
